# Non operative management of postpartum Diastasis Recti: a systematic review and metanalysis of randomized controlled trials

**DOI:** 10.1007/s10029-026-03671-1

**Published:** 2026-04-17

**Authors:** S. Capoccia Giovannini, H. Hoffmann, U. Bracale, G. Cavallaro, B. M. Iacone, G. Camerini, C. Stabilini

**Affiliations:** 1 Department of Integrated Surgical and Diagnostic Sciences, University of Genoa, Genoa, Italy; 2 Zwei Chirurgen GmbHCenter for Hernia Surgery and Proctology, St. Johanns-Vorstadt 44, 4056 Basel, Switzerland; 3 Department of Medicine, Surgery and Dentistry, University of Salerno. General and Emergency Surgical Unit AOU San Giovanni E Ruggi D’Aragona- Salerno, Salerno, Italy; 4 Department of Surgery, Sapienza University of Rome, Rome, Italy

**Keywords:** Postpartuum diastasis recti, Non operative management, Core exercise, Rectus abdominis diastasis

## Abstract

**Purpose:**

Rectus Abdominis Diastasis (RAD) is a prevalent postpartum condition, yet consensus regarding the efficacy of conservative management remains limited. This meta-analysis evaluates the effectiveness of structured exercise programs in reducing Inter-Recti Distance (IRD) and improving functional outcomes in postpartum women compared to no-treatment or standard care.

**Methods:**

A systematic search was conducted for Randomized Controlled Trials (RCTs) published before August 2025. The primary outcome was IRD reduction; the secondary outcome was physical disability assessed via the Oswestry Disability Index (ODI). Methodological quality and certainty of evidence were evaluated using Revised Cochrane Risk-of-Bias (RoB 2) and GRADE criteria.

**Results:**

Nine RCTs (450 participants) were included for IRD analysis, demonstrating a significant reduction in the exercise group (MD: -8.05 mm; 95% CI: -10.43, -5.68; *p* < 0.05). Subgroup analyses showed that interventions initiated < 3 months postpartum achieved greater reduction (MD: -10.2 mm; 95% CI: -14.94, -5.46) than delayed starts. Crucially, while no significant difference was found between specific types of training (*p* = 0.32), a consistent advantage was observed for structured exercise over no intervention or standard care. Regarding functional outcomes, meta-analysis of 3 comparisons from 2 RCTs (*n* = 115) using the ODI score showed no significant difference between groups (MD: 0.82 higher score; 95% CI: -2.75, 4.38; *p* = 0.75; I^2^0%).

**Conclusions:**

Structured exercise programs significantly reduce IRD in women with RAD. However, this anatomical improvement does not translate into superior functional recovery, as measured by the ODI score, within the observed periods. Further standardized research is warranted to establish optimal clinical protocols and the need for RAD-specific functional scales in future research.

**Supplementary Information:**

The online version contains supplementary material available at 10.1007/s10029-026-03671-1.

## Background

Rectus Abdominis Diastasis (RAD) is a highly prevalent condition, with reported rates ranging from 30 to 70% during pregnancy and persisting in up to 60% of women in the postpartum period [1–3]. Anatomically, it is characterized by the pathological separation of the two rectus abdominis muscle along the linea alba, a condition that often results in a significant impairment of women’s health-related quality of life [4–7].

According to the 2021 European Hernia Society (EHS) guidelines, RAD is clinically defined as a widening and thinning of the Linea alba exceeding 2 cm. This separation is typically assessed via clinical palpation, ultrasound, or caliper measurement, standardized at 3 cm above the umbilicus [8, 9]. Clinically, RAD is frequently associated with compromised core stability, lumbopelvic pain, functional limitations, and decreased aesthetic satisfaction [3, 10]. Consequently, there is a high demand for therapeutic interventions, often including surgical repair, even in cases of mild diastasis where no ventral hernia is present or where the functional deficit is not immediately apparent.

Management strategies encompass both surgical and non-surgical approaches, both aimed at reducing the inter-rectus distance (IRD) and restoring the structural integrity of the abdominal wall. While several surgical techniques—ranging from open plication to minimally invasive procedures involving mesh reinforcement—have been described, a global consensus on the gold-standard surgical approach remains elusive [11–14]. Furthermore, selecting the optimal surgical procedure requires a multifaceted evaluation of the IRD width, concomitant hernias, and skin laxity. Given that many patients are young women of childbearing age who may consider future pregnancies, the decision-making process should carefully weigh patient-centered factors, including the potential for future pregnancies and the preference for minimally invasive options where clinically appropriate [15–17].

In this clinical context, physiotherapeutic programs represent a critical, non-invasive starting point in the treatment pathway. Conservative strategies, primarily targeted physical exercise and abdominal support devices, have been investigated as alternatives to surgical intervention. Although recent Randomized Controlled Trials (RCTs) have reported encouraging results regarding the efficacy of physiotherapy in reducing IRD and improving functional outcomes, the literature is characterized by significant heterogeneity. Variabilities in exercise protocols, measurement methodologies, intervention timing, and patient selection criteria have prevented the establishment of a standardized rehabilitative consensus.

Therefore, the aim of the present meta-analysis is to systematically evaluate the role of structured physical therapy programs in reducing IRD among postpartum women presenting with RAD compared to no-treatment controls, providing evidence-based insights into the efficacy of conservative management.

## Material & method

### Study design and registration

This systematic review and meta-analysis were conducted in accordance with the PRISMA (Preferred Reporting Items for Systematic Reviews and Meta-Analyses) guidelines [18].

The study aimed to evaluate the effectiveness of structured exercise interventions versus control conditions in reducing Inter-Recti Distance and improving clinical outcomes in postpartum women. The present Review was registered on PROSPERO database with number PROSPERO 2026 CRD420261288804.

### Search strategy and selection criteria

A systematic search was performed across major medical databases (PubMed and Scopus) for RCTs published in the English language, without time restriction. The initial search was conducted in January 2024 and subsequently updated in August 2025 (see supplementary file 1). To identify additional pertinent studies, a cross-reference search was conducted by examining the bibliographies of the included articles and their related publications.

The eligibility criteria were defined using the PICO framework:

- Population: Postpartum women (up to 12 months after delivery) with a diagnosis of RAD.
- Intervention: Structured abdominal exercise programs (e.g., core stabilization, transversus abdominis training).
- Comparison: No intervention, minimal educational advice, or standard physical activity.
- Outcome: Primary outcome was the change in IRD (measured in mm). Secondary outcomes included changes in quality of life and physical disability.

### Selection and data collection process

All identified studies were imported into Rayyan software for management and screening [19].

The deduplication process was performed within Rayyan, utilizing its automated duplicate identification algorithm followed by a manual verification by two independent reviewers (SCG and BMI) abstracts. Subsequently all studies were screened based on titles and abstract. All relevant full-text articles underwent further review for final selection. Following full-text assessment, the final set of papers suitable for data extraction was included in the analysis. Disagreements between the two reviewers were resolved through consultation with the senior authors (UB, CS, and GC).

### Data extraction and quality assessment

Data were independently extracted by two reviewers (SCG and BMI), focusing on sample size, participant demographics, intervention protocols, and outcome measures. The methodological quality of the included trials was assessed using the Cochrane Risk of Bias tool (RoB 2) [20], evaluating domains such as the randomization process, deviations from intended interventions, and measurement of the outcome.

### Statistical analysis

Meta-analysis was conducted using Review Manager (RevMan) version 5.4. A random-effects model was employed to account for clinical and methodological heterogeneity. For studies where the mean change and standard deviation (SD) were not directly reported, they were calculated using an assumed correlation coefficient (*r* = 0.5) between pre- and post-intervention scores. Statistical heterogeneity was evaluated using the Chi-square test and the I^2^ statistic.

Subgroup analyses were performed based on timing of intervention, parity, and type of comparison.

To maintain methodological rigor and avoid ‘double-counting’ participants from multi-arm trials, the study by Kaya [21] was treated as two independent comparisons (Kaya 1: Exercise vs. Corset; Kaya 2: Exercise + Corset vs. Corset). In accordance with the Cochrane Handbook for Systematic Reviews of Interventions, the shared control group (*n* = 15) was split into two smaller cohorts of 7 and 8 participants, respectively, for the quantitative synthesis. This approach explains the presence of 10 comparisons in the forest plots despite the inclusion of 9 original RCTs.

### Certainty of evidence and summary of findings

The overall certainty of the evidence for the primary and secondary outcomes was assessed using the GRADE (Grading of Recommendations Assessment, Development and Evaluation) approach. The evidence was classified into four levels: high, moderate, low, or very low.

The assessment considered five specific domains: risk of bias, inconsistency (I^2^), indirectness, imprecision (confidence intervals), and publication bias. Summary of Findings (SoF) tables were generated using GRADEpro GDT software to provide a transparent and concise summary of the main results, including the absolute and relative magnitude of the effects and the quality of the evidence for each outcome [22–25].

### Secondary outcomes

Data regarding secondary clinical outcomes, specifically focusing on physical disability and health-related quality of life, were extracted from the included trials. The primary instrument identified for assessing functional impairment was the Oswestry Disability Index (ODI). Other measures reported in a minority of studies included the Pelvic Girdle Pain Questionnaire, the Short Form-36 (SF-36), and the Visual Analogue Scale (VAS).

The Oswestry Disability Index is a validated instrument originally developed by orthopedics to evaluate disability in patients with spinal disorders. First published in 1980, subsequent modifications and versions have broadened its applicability beyond the initial scope. The ODI comprises 10 sections that explore various aspects of a patient’s functional status, including pain intensity, self-care ability, lifting capacity, walking, sitting, standing, sleeping, traveling, social life, and sexual function. Within each section, the patient selects one of six provided statements that most accurately reflects their current condition. Each statement is assigned a progressive score ranging from 0 to 5. The maximum total score achievable is 50, from which the percentage of disability is then calculated [26, 27].

The development of RAD in postpartum women is closely linked to alterations in the pelvic floor musculature and the presence of Diastasis Recti, which together impair the abdominal canister’s ability to distribute intra-abdominal forces effectively [10].

## Results

### Study selection

**Fig. 1:**
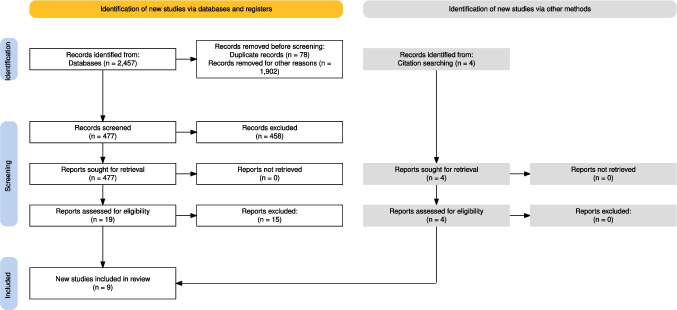
PRISMA 2020 flow diagram

A systematic literature search of the PubMed and Scopus databases initially retrieved 2,457 studies. After screening titles and abstracts, 19 studies were selected for full-text review. Ultimately, 8 RCTs met the inclusion criteria. Four additional RCTs were identified through manual searches of other sources, resulting in a total of 12 potential RCTs. However, three studies [28–30] were excluded due to the impossibility of data extraction, leaving nine RCTs for the final quantitative analysis. Figure 1 shows the PRISMA 2020 flow diagram through the screening process.

The nine included RCTs [21, 31–38] published between 2017 and 2023, collectively encompassed 450 postpartum women: 232 in the intervention arm and 218 in the control group. All studies enrolled participants within 12 months postpartum, with the majority recruited within the first three months. Regarding parity, four studies focused on primiparous women, four included both primiparous and multiparous women, and one did not report parity data. The groups were comparable at baseline, with a mean age of 30.4 ± 2.83 years in the intervention group (Group A) and 30.6 ± 3.6 years in the control group (Group B). The mean Body Mass Index (BMI) was 25.7 ± 2.7 kg/m^2^ and 25.2 ± 3.4 kg/m^2^, respectively.

Definitions and diagnostic thresholds for RAD varied across trials. Eight studies assessed RAD using ultrasound, while one employed a digital caliper. Diagnostic criteria included a separation greater than two finger-widths [31, 36], a threshold exceeding 2.5 cm [21, 33–35], or a threshold of 2 cm [38]. Two studies did not specify a quantitative cutoff for inclusion [32, 37]. Detailed participant characteristics and treatment protocols are summarized in Table 1.

**Table 1:** Included studies data

Study (YEAR)	Participants (N)	Parity	Enrollment (Postpartum)	Treatment vs. Comparison	Frequency & Duration	Follow-up	Baseline IRD (MM) [Mean ± SD]	Post-Treatment IRD (MM) [Mean ± SD]
Gluppe (2023)	70	Mixed	6–12 months	Structured Abdominal Exercise vs. No Intervention	12 weeks (Daily)	12 weeks	A: 37.0 ± 8.0 B: 40.0 ± 10.0	A: 36.0 ± 9.0 B: 38.0 ± 10.0
Thabet (2019)	40	Primiparous	2 months	Deep Core Stability + Trad. Exercise vs. Trad. Exercise	8 weeks (3 sessions/wk)	2 months	A: 28.3 ± 1.0 B: 28.5 ± 0.9	A: 20.0 ± 0.7 B: 23.6 ± 1.1
Peiqin (2022)	66	Mixed	2–6 months	EMG-Biofeedback + NMES + Exercise vs. NMES + Exercise	6 weeks (2 sessions/wk)	1.5 months	A: 28.0 ± 9.0 B: 29.0 ± 7.0	A: 16.0 ± 3.0 B: 20.3 ± 3.0
Kamel (2017)	60	Mixed	2 months	NMES + Abdominal Exercise vs. Abdominal Exercise alone	8 weeks (3 sessions/wk)	2 months	A: 28.7 ± 1.6 B: 28.2 ± 1.2	A: 13.9 ± 1.3 B: 22.8 ± 1.2
Kaya 1 (2023)	30	Mixed	6–12 weeks	Core Stabilization Exercises vs. Abdominal Corset	8 weeks (3 sessions/wk)	2 months	A: 175.0 ± 38.0 B: 158.0 ± 38.0	A: 145.0 ± 40.0 B: 127.0 ± 33.0
Kaya 2 (2023)	30	Mixed	6–12 weeks	Core Stabilization + Abdominal Corset vs. Abdominal Corset	8 weeks (3 sessions/wk)	2 months	A: 187.0 ± 54.0 B: 158.0 ± 38.0	A: 130.0 ± 40.0 B: 127.0 ± 33.0
Shohaimi (2022)	57	Primiparous	6–12 weeks	Home-based STEP Exercise Program vs. Standard Care	8 weeks (3 sessions/wk)	2 months	A: 22.9 ± 5.3 B: 20.3 ± 4.8	A: 16.7 ± 3.6 B: 18.7 ± 5.4
Awad (2021)	50	Mixed	3 months	Progressive Prone Plank Program vs. No Intervention	8 weeks (3 sessions/wk)	2 months	A: 29.7 ± 16.1 B: 29.7 ± 16.1	A: 20.8 ± 2.0 B: 26.1 ± 2.1
El-Mekawy (2013)	30	Primiparous	Immediate (2nd day)	Abdominal Exercise Program vs. Abdominal Supporting Belt	6 weeks (Daily)	1.5 months	A: 30.1 ± 1.2 B: 31.0 ± 1.1	A: 12.2 ± 0.8 B: 24.3 ± 1.5
Wei (2021)	32	Mixed	6 months	Electrical Stimulation + Exercise vs. Exercise alone	6 weeks (3 sessions/wk)	1.5 months	A: 31.0 ± 3.0 B: 29.0 ± 3.0	A: 16.0 ± 3.0 B: 21.0 ± 4.0

#### Interventions

The included trials described various exercise programs for RAD treatment, with durations ranging from six to eight weeks and frequencies of one to three sessions per week. Six studies compared structured exercise modules to no intervention [25–30], while three compared structured programs to "normal" or standard exercise [31–33]. The mean follow-up duration was two months (range: 0–3 months).

#### Risk of bias

**Fig. 2:**
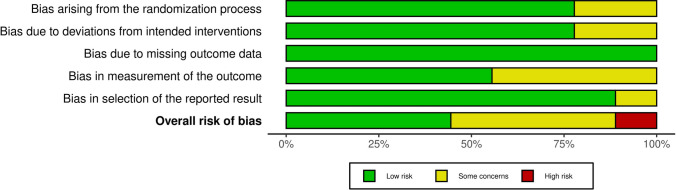
Risk of Bias assessment with ROB2 tool

According to the RoB2 tool evaluation for the IRD reduction outcome, four studies were rated as having a "low" risk of bias, four presented "some concerns," and one was classified as "high risk” as showed in Fig. 2.

#### Meta-analysis of Inter-Recti Distance (IRD) reduction

**Fig. 3:**
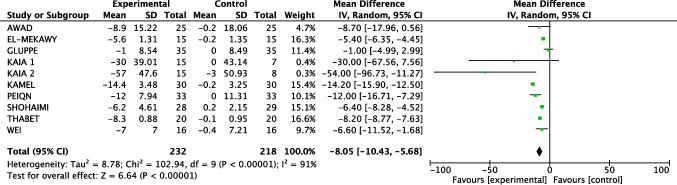
Outcome: IRD reduction

The pooled analysis of nine RCT demonstrated a statistically significant reduction in IRD in the exercise group (232 patients) compared to the control group (218 patients), with a Mean Difference (MD) of −8.05 mm (95% CI: −10.43 to −5.68, *p* < 0.05). Substantial heterogeneity was observed (I^2^ > 91%) (Fig. 3).

#### Subgroup analyses

To explore the observed heterogeneity, several subgroup analyses were performed (see supplementary file):

- **Timing of Intervention:** Exercise was found to be highly effective when initiated in the immediate postpartum period (less than 3 months), showing an IRD reduction of MD −10.2 mm (95% CI: −14.94 to −5.46 mm). In contrast, interventions started after 6 months demonstrated a lower reduction (MD −5.93 mm; 95% CI: −9.93 to −1.93 mm). However, the test for subgroup differences did not reach statistical significance (*p* = 0.18).
- **Parity:** Both primiparous and multiparous women experienced significant reductions in IRD. The pooled MD was −8.65 mm (95% CI: −13.56 to −3.75) for primiparous women and −8.55 mm (95% CI: −15.86 to −1.25 mm) for multiparous/mixed populations. The test for subgroup differences yielded a *p*-value of 0.98, indicating that parity did not significantly influence the intervention’s efficacy in this sample.
- **Training Protocols:** A comparison was made between studies using structured exercise programs versus standard exercise and those comparing exercise versus no intervention. Structured programs vs standard exercise demonstrated a more robust IRD reduction (MD −9.59 mm; 95% CI: −13.45 to −5.72 mm) compared to studies with a no-intervention control (MD −6.87 mm; 95% CI: −10.51 to −3.23 mm). Nevertheless, the test for subgroup differences was not significant (*p* = 0.32), suggesting that the specific comparison model was not a primary determinant of clinical success.

The impact of exercise on functional recovery was assessed via the Oswestry Disability Index. A meta-analysis of 3 comparisons from 2 RCTs (*n* = 115) revealed no significant difference between the exercise and control groups (MD: 0.82; 95% CI: −2.75, 4.38; *p* = 0.75) with a remarkably high consistency across trials. Additional outcomes, including SF-36 scores and VAS pain scales, are summarized in Table 2.

**Table 2:** Summary of quality of life and functional outcomes

Study	Measure	Key Findings
Gluppe (2023)	ODI	No significant difference; minimal baseline disability
Thabet (2019)	QoL	Significant improvement in the deep core stability group
Peiqin (2022)	SF-36	Significant QoL increase with multimodal therapy
Kaya (2023)	ODI, VAS	Favorable trend in pain reduction and functional recovery

The main results of these analyses are presented in Table 3 as summary of findings tables produced after evaluation performed according to GRADE methodology.

**Table 3:** Summary of findings table physical therapy compared to control for postoperative rectus diastasis

Certainty assessment							Summary of findings				
Participants (studies) Follow-up	Risk of bias	Inconsistency	Indirectness	Imprecision	Publication bias	Overall certainty of evidence	Study event rates (%)		Relative effect (95% CI)	Anticipated absolute effects	
							With control	With physical therapy		Risk with control	Risk difference with physical therapy
IRD change											
450 (9 RCTs)	serious^a^	not serious	not serious	not serious	none	⨁⨁⨁◯ Moderate^a^	218	232	-	218	MD **8.05 mm lower** (10.43 lower to 5.68 lower)
IRD change—immediate postpartuum											
258 (5 RCTs)	serious^a^	serious^b^	not serious	serious^c^	none	⨁◯◯◯ Very low^a,b,c^	122	136	-	122	MD **10.2 lower** (14.94 lower to 5.46 lower)
IRD change—late postpartuum											
191 (4 RCTs)	serious^a^	serious^b^	not serious	not serious	none	⨁⨁◯◯ Low^a,b^	96	95	-	96	MD **5.93 mm lower** (9.93 lower to 1.93 lower)
IRD change—primiparous											
197 (4 RCTs)	serious^a^	serious^b^	not serious	not serious	none	⨁⨁◯◯ Low^a,b^	99	98	-	99	MD **8.65 lower** (13.56 lower to 3.75 lower)
IRD change—multiparous											
213 (4 RCTs)	serious^a^	serious^b^	not serious	not serious	none	⨁⨁◯◯ Low^a,b^	99	114	-	99	MD **8.55 lower** (15.86 lower to 1.25 lower)
IRD change—exercise vs nothing											
293 (6 RCTs)	serious^a^	serious^b^	not serious	serious^c^	none	⨁◯◯◯ Very low^a,b,c^	139	154	-	139	MD **6.87 lower** (10.51 lower to 3.23 lower)
IRD change – structured exercise vs normal exercise											
157 (3 RCTs)	serious^a^	serious^b^	not serious	not serious	none	⨁⨁◯◯ Low^a,b^	79	78	-	79	MD **9.59 lower** (13.45 lower to 5.72 lower)
Oswestri disability scale											
115 (3 RCTs)	serious^a^	not serious	not serious	very serious^d^	none	⨁◯◯◯ Very low^a,d^	50	65	-	50	MD **0.82 higher** (2.75 lower to 4.38 higher)

*CI* confidence interval, *MD* mean difference

^a^more than 50% of studies are at "some concerns" Risk of bias according ROB2 evaluation

^b^High heterogeneity

^c^wide CI

^d^small sample size

## Discussion

The management of postpartum Rectus Abdominis Diastasis is a clinical challenge, often the patients is advised both a wait-and-see approach from the general practitioner and various surgical procedures by abdominal wall specialists [39]. Our meta-analysis of nine RCTs demonstrates that structured exercise programs lead to a statistically significant reduction in IRD compared to standard care or no intervention (MD: −8.05 mm) and the results are consistent with recommendations of a recent Consensus Conference on post-gravidic RAD [11].

However, a critical finding of this study is an "anatomical-functional dissociation": the significant reduction in IRD did not translate into superior functional recovery as measured by the Oswestry Disability Index. This discrepancy suggests that clinical recovery and perceived disability may follow different temporal trajectories or be influenced by the high baseline functional status of the participants. However, the observed reduction in IRD could be considered clinically relevant, especially when adopting the EHS definition of RAD from 20 mm separation even the functional results are inconsistently reported. Nevertheless, our subgroup analysis suggests the existence of a "golden window" for conservative treatment. Interventions proposed within the first three months after delivery, achieved a greater reduction (−10.02 mm) than those started later. This could be hypothetically connected to the concept of a higher myofascial plasticity connected to hormonal influence (e.g., relaxin) present in the early postpartum period [40], which may facilitate the remodeling of the linea alba under controlled mechanical loading. Furthermore, the clinical significance of IRD reduction must be interpreted with caution. As highlighted by Janes et al. [41], postpartum changes often involve not only the widening of the linea alba but also alterations in the width and quality of the rectus muscle bellies themselves. Patients presenting with a "floppy abdomen" or loss of core stability may have a relatively small IRD if the underlying pathology is related to muscle atrophy or global fascial laxity. Therefore, while our study confirms that exercises can reduce the inter-recti gap, the decision-making process must remain individualized.

The most striking result is that while the IRD narrowed, the perceived disability (ODI scores) remained similar between the exercise and control groups. This discrepancy can be explained by several factors. First, the ODI is not a specific score for RAD and was originally designed for spinal disorders [26]; although validated for low back pain, it may not be sensitive enough to capture the specific core-stability nuances associated with RAD. We argue that future research should prioritize the development and validation of RAD-specific instruments focused on "return to nulliparous activity levels" and aesthetic-functional satisfaction rather than generic pain scales. Second, the "standard care" or "no intervention" groups often experience a degree of natural recovery. Finally, functional recovery in RAD is likely more dependent on the tension-generating capacity of the linea alba and the recruitment of the transversus abdominis than on the absolute distance between the muscle bellies.

We observed substantial statistical heterogeneity (I^2^ > 90%), largely driven by outliers such as the trials by Kaya [21], which reported extreme IRD reductions starting from an exceptionally high baseline IRD values (up to 187 mm), which were verified against the primary source. These variations underscore the lack of a standardized “threshold” for physiotherapy in RAD. While our analysis showed no significant difference between various types of training (e.g., core stability vs. general exercise), structured programs consistently outperformed simple observation. This suggests that the supervision and consistency of the program may be more important than the specific anatomical focus of the exercises. Additionally, the use of abdominal corsets in the Kaya trial provides a mechanical confounder; the corset may physically maintain the abdominal wall in a shortened position, potentially facilitating the remodeling of connective tissue independently of muscle strengthening.

For the abdominal wall surgeon, these results could reinforce the role of physiotherapy as a first-line treatment. If a structured 8-week program can reduce the IRD by nearly 1 cm, patients initially presenting with "moderate" diastasis may be downgraded to "mild" cases, potentially avoiding surgery or simplifying the eventual repair. However, the lack of ODI improvement suggests that if a patient’s primary complaint is functional instability or chronic lumbopelvic pain that fails to respond to exercise despite IRD reduction, the underlying issue may be a loss of fascial integrity that potentially can yield benefits from surgical plication but more research on this subgroup of patients should be conducted. Furthermore, the nutritional and physical status of women post-pregnancy may be altered, potentially introducing confounding biases into the analysis of observed results [42]. Another significant source of bias is the possible presence of undiagnosed ventral hernias, frequently associated with rectus diastasis in the post-pregnancy population; however, they are often not systematically screened for or analyzed in the included trials, which may lead to an underestimation of pre-existing abdominal wall defects [43, 44].

Several are the limitations of the present analysis: the first is a high statistical heterogeneity of our estimates: the effect of some studies [21] suggest either differences in measurement technique (ultrasound vs. caliper) or potential bias in reporting that may skew the values. Moreover, on the side of diagnostic Inconsistency, the included studies used different cut-offs for RAD (2 cm, 2.5 cm, or "two fingers"). This lack of a standardized inclusion threshold means the population is "diluted" with both very mild and very severe cases, which respond differently to exercise. As already mentioned, the use of ODI in the current context can introduce bias since most postpartum women are young and active; their "disability" might be subtle (e.g., difficulty with high-impact exercise or "doming" during core work) which the ODI, focused on severe back pain, might fail to detect. Another relevant limitation is the short follow-up of the presented results (2 months), the lack of long-term data (6–12 months), reduce our certainty on the possibility to observe if the exercise-induced IRD reduction is permanent or if the control group eventually goes through a natural healing process. Both the development and resolution of RAD are significantly modulated by hormonal fluctuations, with physiological recovery typically concluding by 18 months postpartum. Since the majority of the included studies focus exclusively on participants within this timeframe, the observed reduction in IRD may occur as part of a spontaneous involution process, potentially confounding the isolated therapeutic effect of physiotherapy. Nevertheless, as previously discussed, a favorable ‘therapeutic window’ appears to exist during the first postpartum months; the lack of significant progress during this early phase may constrain the potential for further spontaneous improvement. This highlights the importance of timely intervention, although long-term follow-up studies are needed to confirm this hypothesis. Finally, several methodological biases were observed in the analyzed papers: with only four studies rated as "low risk" and others having "some concerns" or "high risk," the GRADE certainty of evidence is likely to be downgraded to “Low," meaning the true effect might be substantially different from what the meta-analysis shows. This significantly reduces the statistical power to detect a real difference in disability.

In conclusion, structured exercise is an effective tool for anatomical narrowing of the Linea alba in the postpartum period, particularly when started early. Nevertheless, clinicians should manage patient expectations, as a narrower IRD does not automatically guarantee a reduction in perceived physical disability in the short term. Future research should focus on developing RAD-specific functional scores and long-term follow-up to determine if early exercise prevents the later development of ventral hernias [45].

## Supplementary Information

Below is the link to the electronic supplementary material.

Supplementary file1 (DOCX 12 KB)

Supplementary file2 (PDF 494 KB)

Supplementary file3 (PDF 470 KB)

Supplementary file4 (PDF 496 KB)

Supplementary file5 (PDF 178 KB)

## Data Availability

All data are available in supplementary material, for any other issue or information please refer to the corresponding author Dr. Capoccia Giovannini (scapocciagiovannini@gmail.com).

## References

[1] Bixo L, Sandblom G, Österberg J, Stackelberg O, Bewö K, Olsson A (2022) Association between inter-recti distance and impaired abdominal core function in post-partum women with diastasis recti abdominis. J Abdom Wall Surg:10909. 10.3389/jaws.2022.1090910.3389/jaws.2022.10909PMC1083164838314149

[2] Cavalli M, Aiolfi A, Bruni PG, Manfredini L, Lombardo F, Bonfanti MT, Bona D, Campanelli G (2021) Prevalence and risk factors for diastasis recti abdominis: a review and proposal of a new anatomical variation. Hernia:883-890. 10.1007/s10029-021-02468-810.1007/s10029-021-02468-8PMC837091534363190

[3] Fernandes da Mota PG, Pascoal AG, Carita AI, Bø K (2015) Prevalence and risk factors of diastasis recti abdominis from late pregnancy to 6 months postpartum, and relationship with lumbo-pelvic pain, Man Ther:200-205. 10.1016/j.math.2014.09.00210.1016/j.math.2014.09.00225282439

[4] Fuentes Aparicio L, Rejano-Campo M, Donnelly GM, Vicente-Campos V (2021) Self-reported symptoms in women with diastasis rectus abdominis: a systematic review, J Gynecol Obstet Hum Reprod:101995. 10.1016/j.jogoh.2020.10199510.1016/j.jogoh.2020.10199533227494

[5] Hagovska M, Dudic R, Dudicova V, Svihra J, Urdzik P (2024) Prevalence of diastasis m. rectus abdominis and pelvic floor muscle dysfunction in postpartum women. Bratisl Lek Listy:12-16. 10.4149/bll_2024_00310.4149/BLL_2024_00338041840

[6] Hagovská M, Dudič R, Švihra J, Urdzík P (2024) Relationships of diastasis recti abdominis with stress urinary incontinence and pelvic floor muscle dysfunction in postpartum women. Eur J Obstet Gynecol Reprod Biol:222-226. 10.1016/j.ejogrb.2024.08.00610.1016/j.ejogrb.2024.08.00639154519

[7] Olsson A, Kiwanuka O, Sandblom G, Stackelberg O (2021) Evaluation of functional outcomes following rectus diastasis repair-an up-to-date literature review. Hernia:905-914. 10.1007/s10029-021-02462-010.1007/s10029-021-02462-0PMC837091834302558

[8] Hernández-Granados P, Henriksen NA, Berrevoet F, Cuccurullo D, López-Cano M, Nienhuijs S, Ross D, Montgomery A (2021) European Hernia Society guidelines on management of rectus diastasis. Br J Surg:1189-1191. 10.1093/bjs/znab12810.1093/bjs/znab128PMC1036486034595502

[9] Tung RC, Towfigh S (2021) Diagnostic techniques for diastasis recti. Hernia:915-919. 10.1007/s10029-021-02469-710.1007/s10029-021-02469-734313855

[10] Gluppe S, Ellstrom Engh M, Kari B (2021) Women with diastasis recti abdominis might have weaker abdominal muscles and more abdominal pain, but no higher prevalence of pelvic floor disorders, low back and pelvic girdle pain than women without diastasis recti abdominis. Physiotherapy:57-65. 10.1016/j.physio.2021.01.00810.1016/j.physio.2021.01.00833691943

[11] Bracale U, Stabilini C, Cavallaro G, Pecchini F, Sarno G, Agresta F, Carlucci M, Rocchetti S, Sartori A, Di Leo A, Andreuccetti J, Pignata G, Tartaglia E, Sagnelli C, Cuccurullo D, Iossa A, Vettoretto N, Lionetti R, Bertoglio C, Confalonieri M, Testini M, Soliani G, Galatioto C, Crucitti A, Piccoli M, Formisano G, Iacone B, Aiolfi A, Procida G, Montori G, Tramontano S, Balla A, Giovannini SC, Cavalli M, Campanelli G, Podda M (2025) The Italian national consensus conference on the diagnosis and treatment of Rectus Abdominis diastasis in Post-gravidic Women. Hernia:213. 10.1007/s10029-025-03403-x10.1007/s10029-025-03403-x40576747

[12] Ferrara F, Fiori F (2024) Laparoendoscopic extraperitoneal surgical techniques for ventral hernias and diastasis recti repair: a systematic review. Hernia:2111-2124. 10.1007/s10029-024-03144-310.1007/s10029-024-03144-3PMC1153049139312025

[13] Lelli G, Iossa A, Angeles FDE, Micalizzi A, Fassari A, Soliani G, Cavallaro G (2025). Mini-invasive surgery for diastasis recti: an overview on different approaches. Minerva Surg:60-75. 10.23736/s2724-5691.24.10587-410.23736/S2724-5691.24.10587-440059604

[14] Mommers EHH, Ponten JEH, Al Omar AK, de Vries Reilingh TS, Bouvy ND, Nienhuijs SW (2017) The general surgeon’s perspective of rectus diastasis. A systematic review of treatment options, Surg Endosc:4934-4949. 10.1007/s00464-017-5607-910.1007/s00464-017-5607-9PMC571507928597282

[15] López-Cano M, Verdaguer Tremolosa M, Hernández Granados P, Pereira JA (2023) Open vs. minimally invasive sublay incisional hernia repair. Is there a risk of overtreatment? EVEREG registry analysis. Cir Esp (Engl Ed):S46-s53. 10.1016/j.cireng.2023.02.01310.1016/j.cireng.2023.02.01337951467

[16] Barra F, Biscaldi E, Scala C, Lagana AS, Vellone VG, Stabilini C, Ghezzi F, Ferrero S (2020) A prospective study comparing three-dimensional rectal water contrast transvaginal ultrasonography and computed tomographic colonography in the diagnosis of rectosigmoid endometriosis Diagnostics (Basel) 10.3390/diagnostics1004025210.3390/diagnostics10040252PMC723600932344709

[17] Ferrero S, Stabilini C, Barra F, Clarizia R, Roviglione G, Ceccaroni M (2021) Bowel resection for intestinal endometriosis. Best Pract Res Clin Obstet Gynaecol:114-128. 10.1016/j.bpobgyn.2020.05.00810.1016/j.bpobgyn.2020.05.00832665125

[18] Page MJ, McKenzie JE, Bossuyt PM, Boutron I, Hoffmann TC, Mulrow CD, Shamseer L, Tetzlaff JM, Akl EA, Brennan SE, Chou R, Glanville J, Grimshaw JM, Hróbjartsson A, Lalu MM, Li T, Loder EW, Mayo-Wilson E, McDonald S, McGuinness LA, Stewart LA, Thomas J, Tricco AC, Welch VA, Whiting P, Moher D (2021) The PRISMA 2020 statement: an updated guideline for reporting systematic reviews. BMJ:n71. 10.1136/bmj.n7110.1136/bmj.n71PMC800592433782057

[19] Ouzzani M, Hammady H, Fedorowicz Z, Elmagarmid A (2016), Rayyan-a web and mobile app for systematic reviews. Syst Rev:210. 10.1186/s13643-016-0384-410.1186/s13643-016-0384-4PMC513914027919275

[20] McGuinness LA, Higgins JPT (2021) Risk-of-bias VISualization (robvis): an R package and Shiny web app for visualizing risk-of-bias assessments. Res Synth Methods:55-61. 10.1002/jrsm.141110.1002/jrsm.141132336025

[21] Kaya AK, Menek MY (2023) Comparison of the efficiency of core stabilization exercises and abdominal corset in the treatment of postpartum diastasis recti abdominis. Eur J Obstet Gynecol Reprod Biol:24-30. 10.1016/j.ejogrb.2023.03.04010.1016/j.ejogrb.2023.03.04037031572

[22] Guyatt G, Oxman AD, Sultan S, Brozek J, Glasziou P, Alonso-Coello P, Atkins D, Kunz R, Montori V, Jaeschke R, Rind D, Dahm P, Akl EA, Meerpohl J, Vist G, Berliner E, Norris S, Falck-Ytter Y, Schünemann HJ (2013) GRADE guidelines: 11. Making an overall rating of confidence in effect estimates for a single outcome and for all outcomes, J Clin Epidemiol:151-157. 10.1016/j.jclinepi.2012.01.00610.1016/j.jclinepi.2012.01.00622542023

[23] Guyatt GH, Oxman AD, Santesso N, Helfand M, Vist G, Kunz R, Brozek J, Norris S, Meerpohl J, Djulbegovic B, Alonso-Coello P, Post PN, Busse JW, Glasziou P, Christensen R, Schünemann HJ (2013) GRADE guidelines: 12. Preparing Summary of Findings tables—binary outcomes, J Clin Epidemiol:158-172. 10.1016/j.jclinepi.2012.01.01210.1016/j.jclinepi.2012.01.01222609141

[24] Guyatt GH, Thorlund K, Oxman AD, Walter SD, Patrick D, Furukawa TA, Johnston BC, Karanicolas P, Akl EA, Vist G, Kunz R, Brozek J, Kupper LL, Martin SL, Meerpohl JJ, Alonso-Coello P, Christensen R, Schunemann HJ (2013) GRADE guidelines: 13. preparing summary of findings tables and evidence profiles—continuous outcomes. J Clin Epidemiol:173-183. 10.1016/j.jclinepi.2012.08.00110.1016/j.jclinepi.2012.08.00123116689

[25] Stabilini C, Antoniou S, Berrevoet F, Boermeester M, Bracale U, de Beaux A, East B, Gök H, Lopez Cano M, Muysoms F, Capoccia Giovannini S, Simons M (2024) Engine-an EHS project for future guidelines. J Abdom Wall Surg:13007. 10.3389/jaws.2024.1300710.3389/jaws.2024.13007PMC1127245139071940

[26] Fairbank JC, Couper J, Davies JB, O’Brien JP (1980) The Oswestry low back pain disability questionnaire. Physiotherapy:271–273. 6450426

[27] Fairbank JCT, Pynsent PB (2000) The Oswestry disability index. Spine:2940-2953. 10.1097/00007632-200011150-00017 11074683

[28] Depledge J, McNair P, Ellis R (2023). The effect of Tubigrip and a rigid belt on rectus abdominus diastasis immediately postpartum: a randomised clinical trial. Musculoskelet Sci Pract:102712. 10.1016/j.msksp.2022.10271210.1016/j.msksp.2022.10271236577592

[29] Gluppe SL, Hilde G, Tennfjord MK, Engh ME, Bo K (2018) Effect of a postpartum training program on the prevalence of diastasis recti abdominis in postpartum primiparous women: a randomized controlled trial, Phys Ther:260-268. 10.1093/ptj/pzy00810.1093/ptj/pzy008PMC596330229351646

[30] Keshwani N, Mathur S, McLean L (2021) The impact of exercise therapy and abdominal binding in the management of diastasis recti abdominis in the early post-partum period: a pilot randomized controlled trial, Physiother Theory Pract:1018-1033. 10.1080/09593985.2019.167520710.1080/09593985.2019.167520731642725

[31] Awad E, Mobark A, Zidan AA, Hamada HA, Shousha TM Effect of progressive prone plank exercise program on diastasis of rectus abdominis muscle in postpartum women: a randomized controlled trial, 10.14198/jhse.2021.16.Proc2.24

[32] El-Mekawy HS, ElDeeb AM, Lythy MAE, Elbegawy AF (2013) Effect of abdominal exercises versus abdominal supporting belt on post-partum abdominal efficiency and rectus separation. Int J Med Health Sci:75–79.

[33] Gluppe SB, Ellstrom Engh M, Bo K (2023) Curl-up exercises improve abdominal muscle strength without worsening inter-recti distance in women with diastasis recti abdominis postpartum: a randomised controlled trial. J Physiother:160-167. 10.1016/j.jphys.2023.05.01710.1016/j.jphys.2023.05.01737286390

[34] Kamel DM, Yousif AM (2017) Neuromuscular electrical stimulation and strength recovery of postnatal diastasis recti abdominis muscles, Ann Rehabil Med:465-474. 10.5535/arm.2017.41.3.46510.5535/arm.2017.41.3.465PMC553235328758085

[35] Liang P, Liang M, Shi S, Liu Y, Xiong R (2022) Rehabilitation programme including EMG-biofeedback- assisted pelvic floor muscle training for rectus diastasis after childbirth: a randomised controlled trial. Physiotherapy:16-21. 10.1016/j.physio.2022.05.00110.1016/j.physio.2022.05.00136219918

[36] Shohaimi S, Husain NRN, Zaki FM, Atan IK (2023) Split tummy exercise program for reducing diastasis recti in postpartum primigravidae: a randomized controlled trial, Korean J Fam Med:102-108. 10.4082/kjfm.22.003510.4082/kjfm.22.0035PMC1004026836966740

[37] Thabet AA, Alshehri MA (2019). Efficacy of deep core stability exercise program in postpartum women with diastasis recti abdominis: a randomised controlled trial. J Musculoskelet Neuronal Interact:62–68. PMC6454249 30839304

[38] Wei R, Yu F, Ju H, Jiang Q (2022) Effect of electrical stimulation followed by exercises in postnatal Diastasis Recti abdominis via MMP2 gene expression Cell Mol Biol (Noisy-le-grand):82-88. 10.14715/cmb/2021.67.5.1210.14715/cmb/2021.67.5.1235818210

[39] Stabilini C, Capoccia Giovannini S, Campanelli G, Cavallaro G, Bracale U, Soliani G, Pecchini F, Frascio M, Carlini F, Longo G, Rubartelli A, Camerini G (2025). Complex abdomen: a scoping review, Hernia:90. 10.1007/s10029-025-03270-610.1007/s10029-025-03270-639928076

[40] Coldron Y, Stokes MJ, Newham DJ, Cook K (2008) Postpartum characteristics of rectus abdominis on ultrasound imaging Man Ther:112-121. 10.1016/j.math.2006.10.00110.1016/j.math.2006.10.00117208034

[41] Janes LE, Fracol ME, Dumanian GA (2019) Appreciation of postpartum changes of the rectus muscles in primary and repeated abdominoplasty. Plast Reconstr Surg:197e-204e. 10.1097/PRS.000000000000586210.1097/PRS.000000000000586231348338

[42] Huisman MG, Veronese G, Audisio RA, Ugolini G, Montroni I, de Bock GH, van Leeuwen BL, PREOP study group (2016) Poor nutritional status is associated with other geriatric domain impairments and adverse postoperative outcomes in onco-geriatric surgical patients - a multicentre cohort study. Eur J Surg Oncol:1009-1017. 10.1016/j.ejso.2016.03.00510.1016/j.ejso.2016.03.00527157495

[43] Capoccia Giovannini S, Vierstraete M, Frascio M, Camerini G, Muysoms F, Stabilini C (2025) Systematic review and meta-analysis on robotic assisted ventral hernia repair: the ROVER review. Hernia:95. 10.1007/s10029-025-03274-210.1007/s10029-025-03274-239966282

[44] Stabilini C, Garcia-Urena MA, Berrevoet F, Cuccurullo D, Capoccia Giovannini S, Dajko M, Rossi L, Decaestecker K, Lopez Cano M (2022). An evidence map and synthesis review with meta-analysis on the risk of incisional hernia in colorectal surgery with standard closure. Hernia:411-436. 10.1007/s10029-021-02555-w10.1007/s10029-021-02555-w35018560

[45] Vierstraete M, Simons M, Borch K, de Beaux A, East B, Reinpold W, Stabilini C, Muysoms F (2022) Description of the Current Da Vinci((R)) training pathway for robotic abdominal wall surgery by the European Hernia Society. J Abdom Wall Surg :10914. 10.3389/jaws.2022.1091410.3389/jaws.2022.10914PMC1083168438314150

