# Supplementary material for: Non operative management of postpartum Diastasis Recti: a systematic review and metanalysis of randomized controlled trials
Source: Hernia. 2026 Apr 17;30(1):164. doi: 10.1007/s10029-026-03671-1 (PMC13090193; doi:10.1007/s10029-026-03671-1)
Supplement: Supplementary file 1 — Supplementary file1 (DOCX 12 KB) [file 10029_2026_3671_MOESM1_ESM.docx]

Search string for systematic review

(((abdominal rectus diastasis) OR (rectus diastasis)) OR (rectus abdominis divarication)) OR (postpartum diastasis)

Limits:

english language

RCT

No time limits applied
