# Supplementary figures and images for: Non operative management of postpartum Diastasis Recti: a systematic review and metanalysis of randomized controlled trials
Source: Hernia. 2026 Apr 17;30(1):164. doi: 10.1007/s10029-026-03671-1 (PMC13090193; doi:10.1007/s10029-026-03671-1)

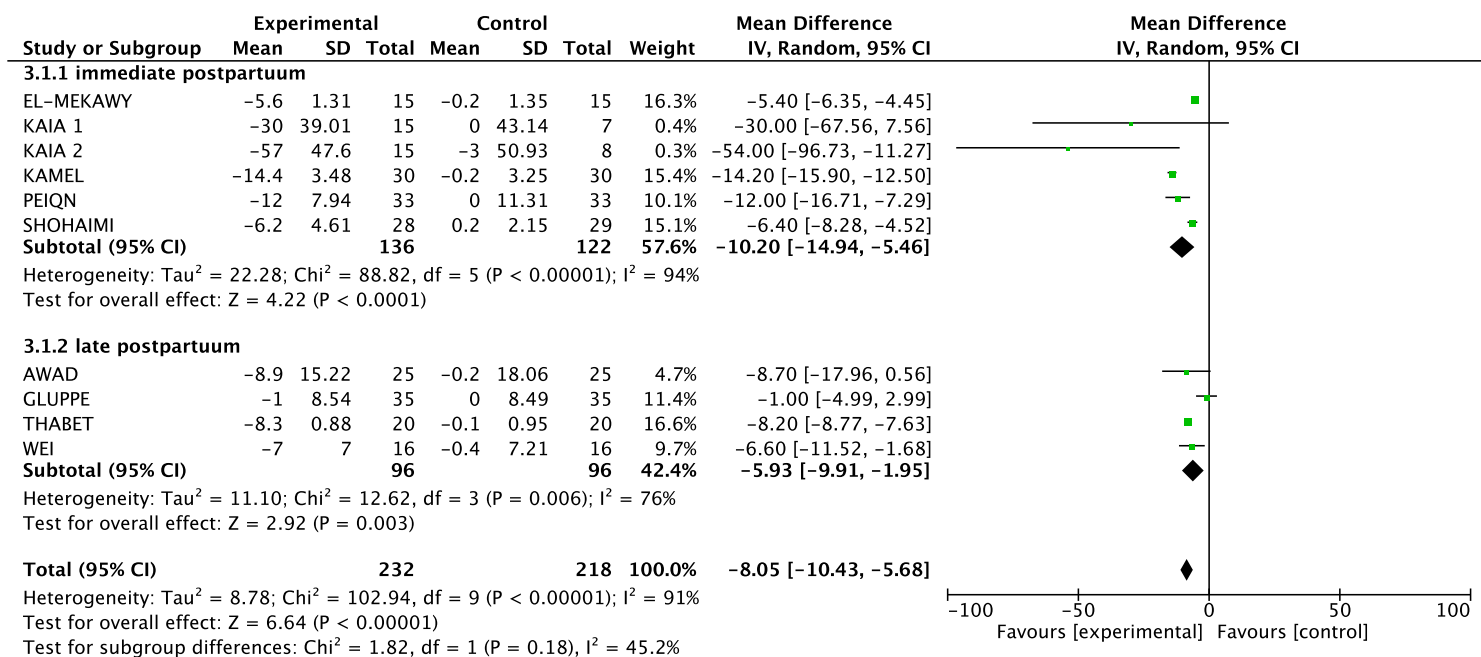

Supplement: Supplementary file 2 — Supplementary file2 (PDF 494 KB) [file 10029_2026_3671_MOESM2_ESM.pdf]

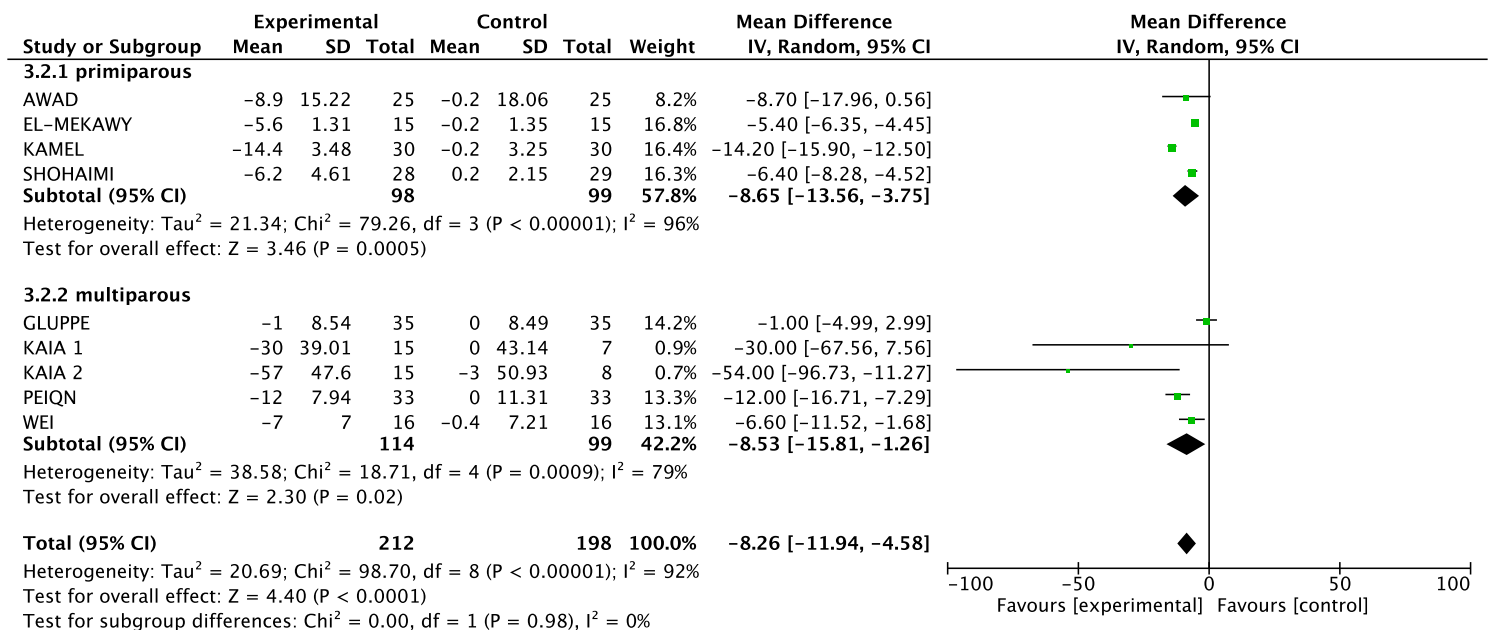

Supplement: Supplementary file 3 — Supplementary file3 (PDF 470 KB) [file 10029_2026_3671_MOESM3_ESM.pdf]

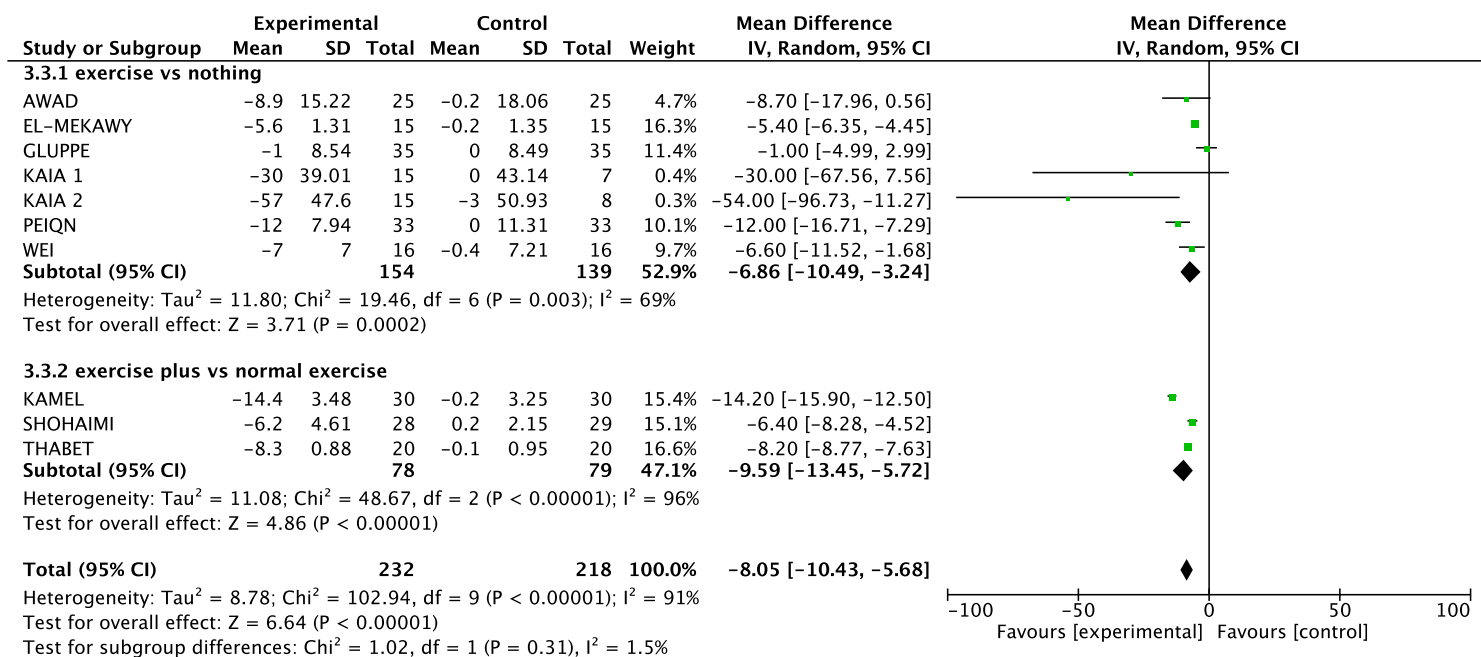

Supplement: Supplementary file 4 — Supplementary file4 (PDF 496 KB) [file 10029_2026_3671_MOESM4_ESM.pdf]

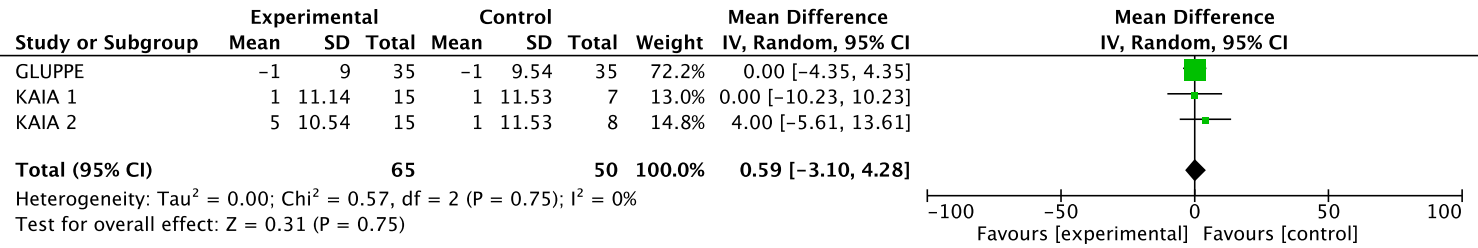

Supplement: Supplementary file 5 — Supplementary file5 (PDF 178 KB) [file 10029_2026_3671_MOESM5_ESM.pdf]
